# Supplementary material for: Quantum verification of NP problems with single photons and linear optics
Source: Light Sci Appl. 2021 Aug 18;10:169. doi: 10.1038/s41377-021-00608-4 (PMC8373877; doi:10.1038/s41377-021-00608-4)
Supplement: Supplementary file 1 — Supplementary Information [file 41377_2021_608_MOESM1_ESM.pdf]

Supplementary Information for "Quantum verification of NP problems with single photons and linear optics"

Aonan Zhang,[1,2] Hao Zhan,[1,2] Junjie Liao,[1,2] Kaimin Zheng,[1,2] Tao Jiang,[1,2] Minghao Mi,[1,2] Penghui Yao,[3,*] and Lijian Zhang[1,2,†]

[1] *National Laboratory of Solid State Microstructures, Key Laboratory of Intelligent Optical Sensing and Manipulation (Ministry of Education) and College of Engineering and Applied Sciences, Nanjing University, Nanjing 210093, China*
[2] *Collaborative Innovation Center of Advanced Microstructures, Nanjing University, Nanjing 210093, China*
[3] *State Key Laboratory for Novel Software Technology, Nanjing University, Nanjing 210093, China*

I. EXPERIMENTAL DETAILS

Photon source. The photon pairs generated by the SPDC source (denoted as signal and idler modes) were coupled into single mode fibers respectively. To characterize the second order coherence value $g^{(2)}(0)$ of heralded single photons, we detect photons with a single-photon avalanche diode in the idler mode (denoted as mode H), of which a click heralds a single photon in the signal mode. The signal mode is split into two modes (labelled as a and b) by applying a half-wave plate set in 22.5° followed by a polarizing beam-splitter. The two modes are also detected by two single-photon avalanche diodes. We register the two-fold and three-fold coincidences between the detectors a, b and H, then the second order coherence value can be calculated by

$$g^{(2)}(0) = \frac{C_{a,b,H}}{C_{a,H}C_{b,H}} N_H \tag{S1}$$

Here $C_{a,b,H}$ denotes the three-fold coincidence rate between detectors a, b and heralding, while $C_{a,H}$ ($C_{b,H}$) denotes the coincidence rate between detectors a (b) and heralding. N_H is the count rate of the heralding. We set the pump power of the source into different levels and measure the second order coherence values, as shown in Fig. S1a. The measured values show an excellent agreement with the linear fitting.

To observe the Hong-Ou-Mandel (HOM) interference between the two photons, we interfere the two photons by a non-polarizing beam-splitter (NPBS) and register the coincidence counts between the two detectors placed in the

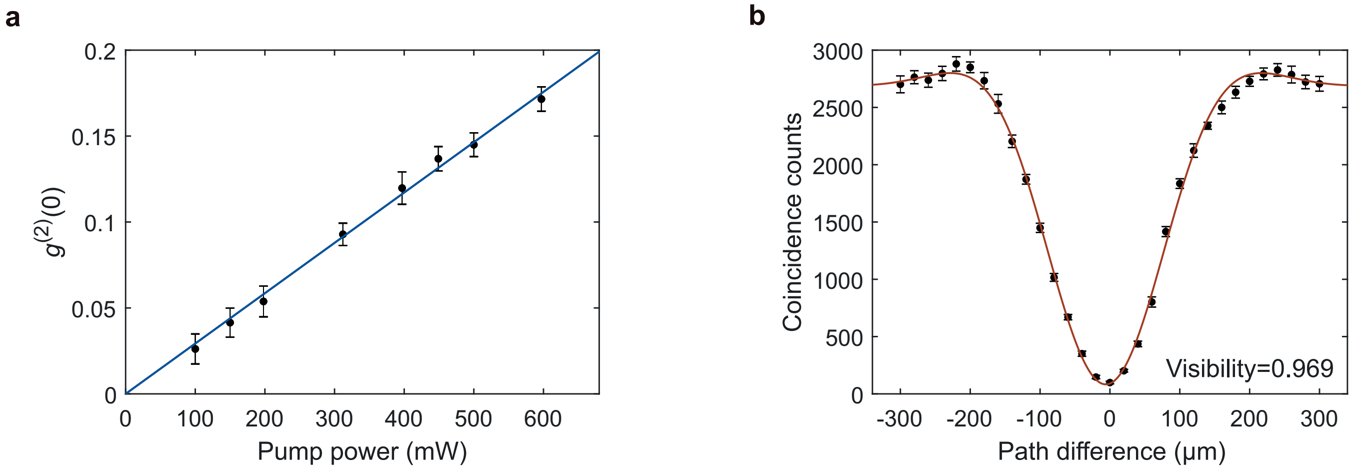

FIG. S1. Characterization of the photon source. (a) Experimental results of the $g^{(2)}$ measurements under different pump powers. The solid line (blue) is the linear curve fitting to the data. The errorbars are the standard uncertainties over 30 runs of the experiment. (b) The Hong-Ou-Mandel interference between the two photons. The solid line (red) is the curve fitting of the data to a Gaussian multiplied by sinc function. The errorbars are the standard uncertainties over 30 runs of the experiment.

* pyao@nju.edu.cn
† lijian.zhang@nju.edu.cn

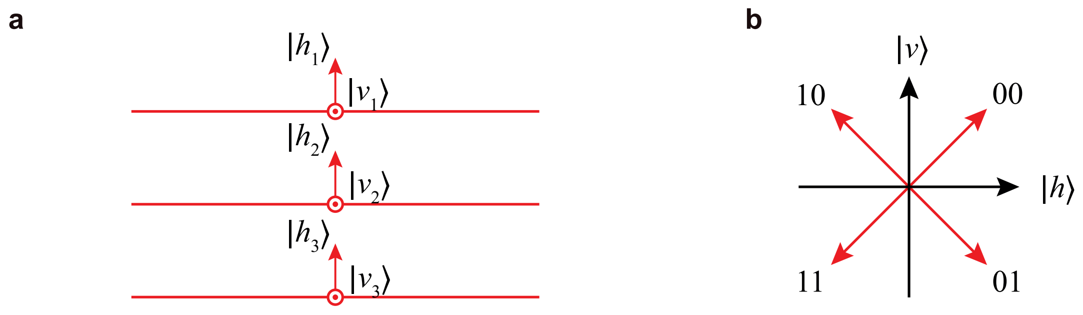

FIG. S2. (a) The mode mapping in the experiment. The horizontal and vertical polarizations in the three path modes $\{|h_1\rangle, |v_1\rangle, |h_2\rangle, |v_2\rangle, |h_3\rangle, |v_3\rangle\}$ correspond to the basis $\{|1\rangle, |2\rangle, |3\rangle, |4\rangle, |5\rangle, |6\rangle\}$ for the proof state. (b) The encoding in the polarization of a single path. The four polarization states correspond to the four possible values 00,01,10 and 11.

two output sides. The experimental results are shown in Fig. S1b and the visibility of the HOM interference is $\mathcal{V} = 0.969 \pm 0.004$.

Proof encoding. In the experiment, the proof is encoded in the polarization and path degrees of freedom of the photon. Each copy of the proof state is a superposition on 3 path modes × 2 polarization modes, which forms a 6-dimensional quantum state (see Fig. S2). The optical modes $\{|h_1\rangle, |v_1\rangle, |h_2\rangle, |v_2\rangle, |h_3\rangle, |v_3\rangle\}$ correspond to the basis of the proof state $\{|1\rangle, |2\rangle, |3\rangle, |4\rangle, |5\rangle, |6\rangle\}$. Here $|h_1\rangle$ ($|v_1\rangle$) denotes the horizontal (vertical) polarization in path 1. Figure S3 shows the experimental implementation of the circuit operations on the six optical modes. The optics axis of each wave plate is pre-calibrated to guarantee accurate encoding and manipulation on the polarization in the following. The detailed procedure of proof encoding includes:

(1) Prepare the photons in a coherent superposition of three path modes with equal amplitudes. The manipulation of path modes is achieved by combining half-wave plates to adjust the polarization and beam-displacers (BDs) that maps the polarization modes to path modes by moving the horizontally polarized photon with a 4 mm lateral displacement. In particular, we set the polarization of the single input path mode to $(\sqrt{2}|h_1\rangle + |v_1\rangle)/\sqrt{3}$ with a half-wave plate, then displace the horizontal polarization into another path mode. The polarization of the path mode is set into $(|h_1\rangle + |v_1\rangle)/\sqrt{2}$ and we displace the horizontal polarization with another BD to realize the superposition of three path modes.

(2) Initialize the phase differences between the three path modes to zero. This is achieved by adjusting the tilt of each beam-displacer and confirmed by monitoring the interference between different path modes with a classical laser light.

(3) After passing the splitting module, the single photonic state undergoes a combination of three unitary transformations $u_j(\theta_j)$. The unitary transformations are implemented by three half-wave plates with electronically-controlled rotation stages (Newport PR50PP). Each wave plate is configured into one of the four angles ($-67.5°, -22.5°, 22.5°, 67.5°$ or $-22.5°, 22.5°, 67.5°, 112.5°$) to realize one of the four sub-operations

$$\frac{1}{\sqrt{2n}}\begin{pmatrix}-1 & -1\\ -1 & 1\end{pmatrix}, \frac{1}{\sqrt{2n}}\begin{pmatrix}1 & -1\\ -1 & -1\end{pmatrix}, \frac{1}{\sqrt{2n}}\begin{pmatrix}1 & 1\\ 1 & -1\end{pmatrix}, \frac{1}{\sqrt{2n}}\begin{pmatrix}-1 & 1\\ 1 & 1\end{pmatrix}$$

Each sub-operation prepares a certain polarization corresponding to the encoding of two variables (see Fig. S2b). For example, the polarization states $\{(|h_1\rangle + |v_1\rangle)/\sqrt{2}, (|h_1\rangle - |v_1\rangle)/\sqrt{2}, (-|h_1\rangle + |v_1\rangle)/\sqrt{2}, (-|h_1\rangle - |v_1\rangle)/\sqrt{2}\}$ correspond to the encoding of the assignments 00,01,10,11 into the subspace of the proof state respectively. Consequently, the three reconfigurable wave plates enable the encoding of all the 64 possible assignments into the proof state (i.e., the 64 proper states). Note the proof state can also be prepared into improper states by setting the wave plates in the proof encoding stage into other angles.

Arthur's computation machine. For each copy of the proof state, Arthur firstly performs the tunable permutations on the optical modes in Stage (ii). As explained in the main text, each module of tunable permutation is composed of a layer of unitary transformations $\{u_j(\theta_j)\}$ and a mode routing P. In our experiment, the unitary transformations can be set into different operations such as

$$Z = \frac{1}{\sqrt{n}}\begin{pmatrix}1 & 0\\ 0 & -1\end{pmatrix}, H = \frac{1}{\sqrt{2n}}\begin{pmatrix}1 & 1\\ 1 & -1\end{pmatrix}, X = \frac{1}{\sqrt{n}}\begin{pmatrix}0 & 1\\ 1 & 0\end{pmatrix}$$

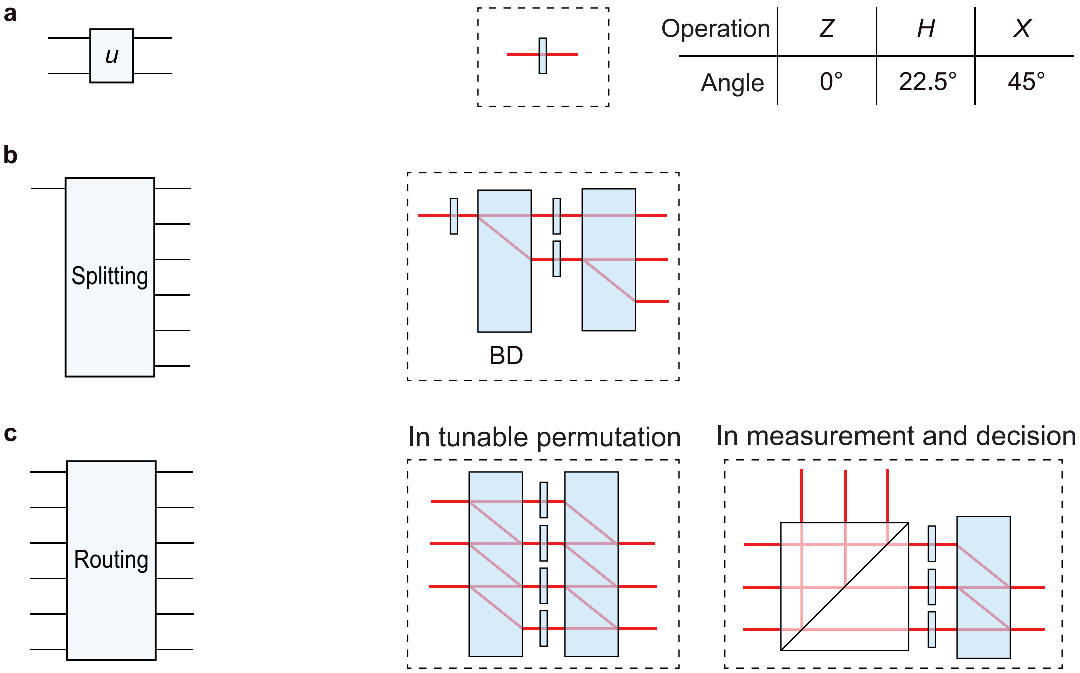

FIG. S3. The correspondence between the circuit operations and the experimental setups. (a-c) The experimental implementations of the unitary transformation, the mode splitting and the mode routing.

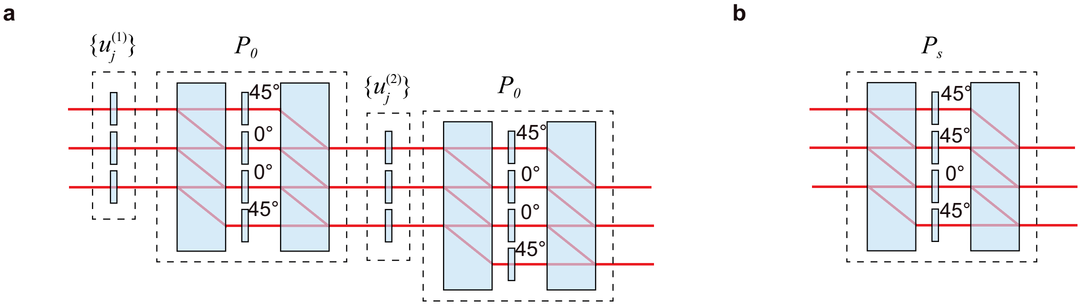

FIG. S4. The detailed settings of the mode routing in the tunable permutations stage. (a) The stage consists of two modules, each with a layer of unitary transformations $\{u_j(\theta_j)\}$ and a mode routing. For most of the cases in our experiments, the two mode routing modules are set into P_0. (b) For the case of verifying the clause $(1, 2, 5, 6)$, the first mode permutation is tuned to P_s by changing the operations of one of the wave plates.

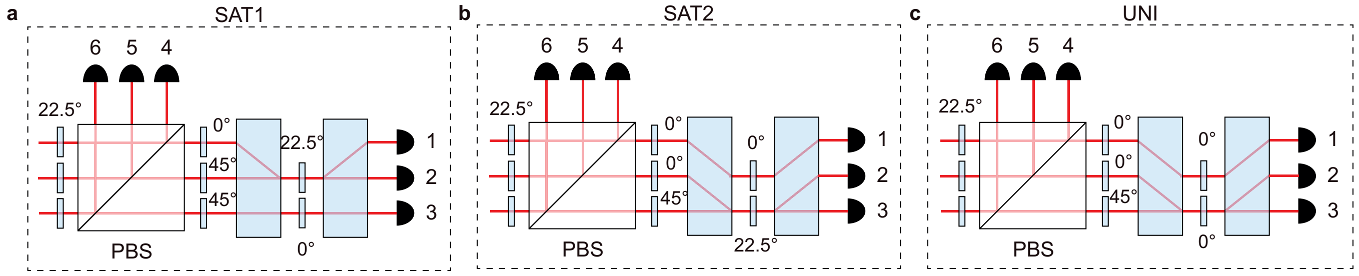

FIG. S5. The detailed settings of the measurement and decision stage. (a) The settings of the wave plates to realize the measurement "SAT1", which performs projection on the modes (1,2,3,4) after the permutation. (b) The settings of the wave plates to realize the measurement "SAT2", which performs projection on the modes (3,4,5,6) after the permutation. (c) The settings of the wave plates to realize the measurement "UNI", which performs interference between the pairs of optical modes after the permutation.

by different settings of the wave plates (see Fig. S3a). Regarding the mode routing (see Fig. S3c, the first box), we can set the wave plates in each module to realize one of the two permutation matrices

$$P_0 = \begin{pmatrix} 0 & 1 & 0 & 0 & 0 & 0 \\ 0 & 0 & 0 & 1 & 0 & 0 \\ 1 & 0 & 0 & 0 & 0 & 0 \\ 0 & 0 & 0 & 0 & 0 & 1 \\ 0 & 0 & 1 & 0 & 0 & 0 \\ 0 & 0 & 0 & 0 & 1 & 0 \end{pmatrix}, P_s = \begin{pmatrix} 0 & 1 & 0 & 0 & 0 & 0 \\ 1 & 0 & 0 & 0 & 0 & 0 \\ 0 & 0 & 0 & 1 & 0 & 0 \\ 0 & 0 & 0 & 0 & 0 & 1 \\ 0 & 0 & 1 & 0 & 0 & 0 \\ 0 & 0 & 0 & 0 & 1 & 0 \end{pmatrix}$$

The configurations of the wave plates for implementing the two permutation matrices are depicted in Fig. S4. Note the operations of the aforementioned two routing modules have negative values -1 in some of the elements in actual implementations, due to the fact that a Pauli-Z sub-operation (a half-wave plate aligned in $0°$) adds a relative π phase in one of the two optical modes. Yet in the following design of configurations we have take the phases added by all the wave plates into consideration to eliminate the effect, therefore the modules equivalently realize the permutations P_0 and P_1. In our experiments, Stage (ii) utilizes two modules of the tunable permutations, as shown in Fig. S4a. The two layers of unitary transformations are denoted as $\{u_j^{(1)}(\theta_j^{(1)})\}$ and $\{u_j^{(2)}(\theta_j^{(2)})\}$ respectively and the overall transformations are thus $U^{(1)} = \bigoplus_{j=1}^{n/2} u_j^{(1)}(\theta_j^{(1)})$ and $U^{(2)} = \bigoplus_{j=1}^{n/2} u_j^{(2)}(\theta_j^{(2)})$. The two mode routing modules are normally set into P_0 for the cases of verifying 14 clauses and all the 15 matchings, whereas the first mode routing is set into P_s for the case of verifying the clause $(1, 2, 5, 6)$. The detailed configurations of waveplates to implement the 15 projections and the 15 matchings are given in Table SI and Table SII respectively.

In Stage (iii), Arthur resorts to one of the three types of measurements depending on which test is chosen and which permutation is performed. The settings of the three types of measurements are depicted in Fig. S5. For all the three types of measurements, the state firstly undergoes a layer of unitary transformations set into H operations and a mode routing (Fig. S3c, the second box). The permutation matrix for this mode routing (in the measurement and decision part) can be described as

$$P_m = \begin{pmatrix} 1 & 0 & 0 & 0 & 0 & 0 \\ 0 & 0 & 1 & 0 & 0 & 0 \\ 0 & 0 & 0 & 0 & 1 & 0 \\ 0 & 1 & 0 & 0 & 0 & 0 \\ 0 & 0 & 0 & 1 & 0 & 0 \\ 0 & 0 & 0 & 0 & 0 & 1 \end{pmatrix}$$

For the satisfiability test, Arthur then performs the measurement "SAT1" or "SAT2", depending on which four variables are verified (upper or lower). For the uniformity test, Arthur performs the measurement "UNI" to interfere the pairs of the optical modes.

Experimental imperfections. In the satisfiability test, each combination of a proof state and a verified clause corresponds to a rejection probability p_c. To characterize the experimental errors, we calculate the average statistical fidelity

$$\mathcal{F}_c = \left(\sqrt{p_c^{\text{the}} p_c^{\text{exp}}} + \sqrt{(1 - p_c^{\text{the}})(1 - p_c^{\text{exp}})} \right)^2 \tag{S2}$$

between the theoretical and experimental projection probabilities (p_c^{the} and p_c^{exp}). The limited interference visibility and the phase fluctuations, together with the systematic errors in the operations, are responsible for the deviations and result in a non-zero projection probability for the satisfying proofs. The alignments of BDs are pre-calibrated to achieve interference visibilities exceeding 99% and the path differences within the interferometers are tuned to zero. Regarding the phase fluctuations, the compact interferometers implemented by the beam displacers (BDs) are stable against environmental perturbations. The paths split by BDs are parallel to each other and the distance between the adjacent paths is a 4 mm lateral displacement, which ensures that the paths undergo nearly the same phase fluctuations. Therefore the phase difference between different paths is passively stabilized. In addition, we built an optical enclosure to shield the experimental set-up from environmental variations to further suppress the phase fluctuations. As a result, the whole set-up can remain stable in a time scale of 3 hours. The systematic errors mainly stem from the misalignments of the wave plates and the beam displacers. In our experiments, the optics axis of each wave plate is calibrated independently with a precision of 0.1°. The repetition errors on the angles of electronically-controlled wave plates (Newport PR50PP) are typically 0.025°. In addition, the unbalanced detection efficiencies for the optical modes may cause deviations of the outcome probabilities. In our experiment we adjust the coupling efficiencies of the optical modes to balance the overall detection efficiencies. It is noteworthy that the scheme

TABLE SI. Detailed configurations of Arthur's setup for the satisfiability test. The rejection mode denotes the optical mode corresponding to the projection on $|c\rangle$, therefore a click on the mode leads to the "reject" decision. Note for the clause $(1, 2, 5, 6)$, the first mode routing in Stage (ii) is set into P_s, whereas for the other 14 clauses the mode routing is set into P_0.

	Clause	Transformations						Measurement	Rejection mode
		$u_1^{(1)}$	$u_2^{(1)}$	$u_3^{(1)}$	$u_1^{(2)}$	$u_2^{(2)}$	$u_3^{(2)}$		
1	(1,2,3,4)	X	X	X	X	X	X	SAT1	2
2	(1,2,3,5)	Z	Z	Z	Z	Z	Z	SAT2	2
3	(1,2,3,6)	Z	Z	X	Z	Z	Z	SAT2	2
4	(1,2,4,5)	X	X	Z	Z	Z	X	SAT2	3
5	(1,2,4,6)	Z	X	X	Z	Z	Z	SAT2	2
6	(1,2,5,6)	X	X	X	X	Z	Z	SAT1	2
7	(1,3,4,5)	Z	X	Z	X	Z	X	SAT2	3
8	(1,3,4,6)	X	X	Z	X	Z	X	SAT1	2
9	(1,3,5,6)	X	Z	X	Z	X	X	SAT2	3
10	(1,4,5,6)	X	X	X	Z	X	X	SAT2	3
11	(2,3,4,5)	X	X	Z	X	Z	X	SAT2	3
12	(2,3,4,6)	X	X	X	X	Z	X	SAT2	3
13	(2,3,5,6)	Z	X	X	Z	Z	Z	SAT1	2
14	(2,4,5,6)	Z	Z	Z	Z	Z	Z	SAT1	1
15	(3,4,5,6)	X	X	X	X	X	X	SAT2	3

TABLE SII. Detailed configurations of Arthur's setup for the uniformity test. For all the 15 matchings the two mode routing modules in Stage (ii) are both set into P_0.

	Matching	Transformations						Measurement
		$u_1^{(1)}$	$u_2^{(1)}$	$u_3^{(1)}$	$u_1^{(2)}$	$u_2^{(2)}$	$u_3^{(2)}$	
1	(1,2),(3,4),(5,6)	X	X	X	X	X	X	UNI
2	(1,2),(3,5),(4,6)	X	Z	X	X	X	Z	UNI
3	(1,2),(3,6),(4,5)	X	Z	Z	X	X	Z	UNI
4	(1,3),(2,4),(5,6)	X	Z	X	Z	X	X	UNI
5	(1,3),(2,5),(4,6)	Z	Z	Z	Z	Z	Z	UNI
6	(1,3),(2,6),(4,5)	Z	Z	X	Z	Z	Z	UNI
7	(1,4),(2,3),(5,6)	X	X	X	Z	X	X	UNI
8	(1,4),(2,5),(3,6)	X	X	Z	Z	Z	X	UNI
9	(1,4),(2,6),(3,5)	Z	X	X	Z	Z	Z	UNI
10	(1,5),(2,3),(4,6)	X	Z	Z	Z	Z	Z	UNI
11	(1,5),(2,4),(3,6)	X	X	Z	Z	Z	Z	UNI
12	(1,5),(2,6),(3,4)	X	X	X	X	Z	X	UNI
13	(1,6),(2,3),(4,5)	X	Z	Z	X	Z	Z	UNI
14	(1,6),(2,4),(3,5)	X	X	Z	X	Z	Z	UNI
15	(1,6),(2,5),(3,4)	X	X	Z	X	Z	X	UNI

only requires at most two layers of cascaded interferometers. Therefore, we expect that the scheme remains a high fidelity even scaled to large size.

For the optical swap test experiments, the main errors include the limited photon indistinguishability from the source part, the non-ideal splitting ratio of the NPBS and the unbalanced detection efficiencies for different detectors. The path differences between the three paths for the two input sides are calibrated by interferometers with classical light.

When scaling the scheme up to higher n, the limited multi-mode interference visibility and HOM visibility would reduce the completeness-soundness gap with the increase of the number of copies K, due to the fact that the completeness is not perfect in practical realizations. However, the QMA(2) protocol allows amplification of success probability by repeating the original protocol, which we demonstrate in Sec. III.

Another factor in practical experiments is the photon loss. The copies of proof states may not be detected by Arthur due to the photon loss in his computation machine. The decision of each test in our experiment is based on the detection of K photons. On average, Merlins need to send $O(K/\eta)$ photons if the overall photon efficiency of Arthur's machine is η. In our experiment, the photon efficiency, accounting for the transmission of the linear optical circuit ($\sim$ 80%), the fiber coupling efficiency ($\sim$ 90%) and the detection efficiency of the SPADs ($\sim$ 60%), is about 43%.

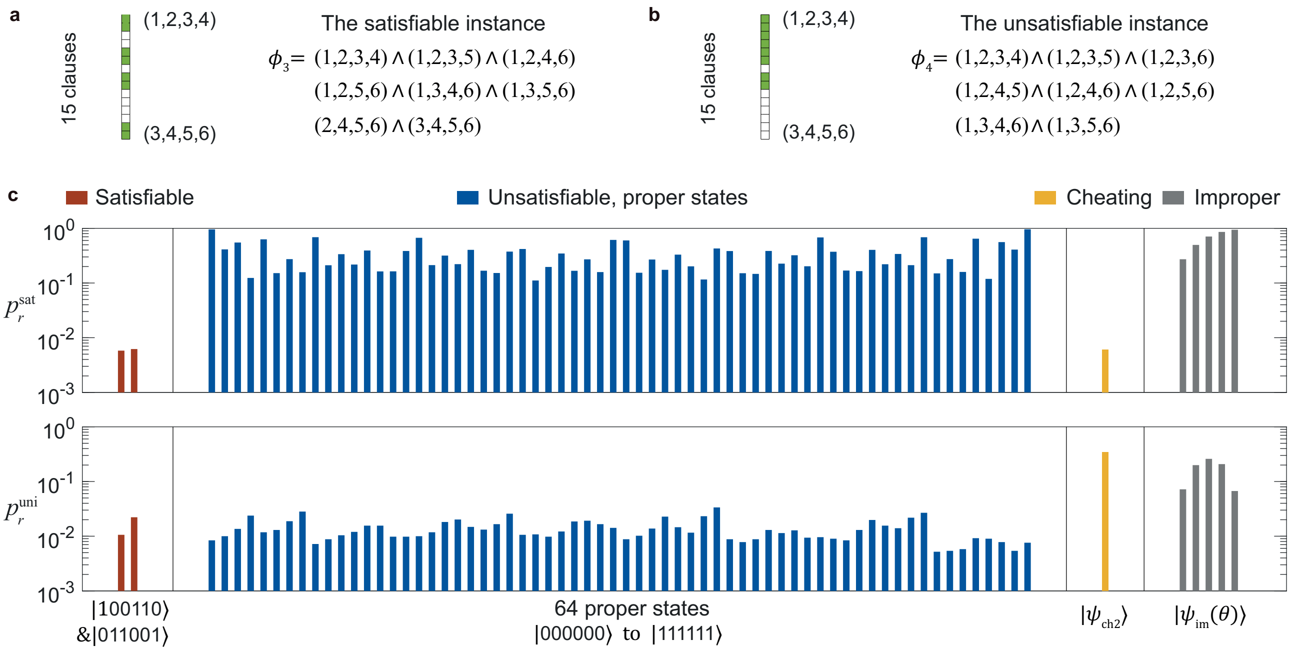

FIG. S6. **Experimental verification of example instances.** **a**, The satisfiable instance ϕ_3. **b**, The unsatisfiable instance ϕ_4. The shaded squares (green) illustrate which 8 of the 15 clauses are chosen in the instance. **c**, The rejection probabilities of the satisfiability test ($p_r^{\rm sat}$, top) and uniformity test ($p_r^{\rm uni}$, down) for different proof states. For the satisfiable instance ϕ_3, Merlins will send states encoding the correct solution thus we show the results for the two satisfying proof states (red bars). For the unsatisfiable instance ϕ_4, we test different cases consisting of sending the 64 proper states (blue bars), a deliberate cheating proof state $|\psi_{\rm ch2}\rangle$ in order to pass the satisfiability test (yellow bars), improper states $|\psi_{\rm im}(\theta)\rangle$ (grey bars). The number of copies $K = 3$ is adopted in the verification.

II. EXPERIMENTAL RESULTS

More examples and detailed results. As a supplement of the experimental results of quantum verification shown in Fig. 3 in the main text, we demonstrate the quantum verification of other example instances, including an unsatisfiable instance where cheating Merlins send copies of $|\psi_{\rm ch2}\rangle$, as shown in Fig. S6. To examine the completeness of the protocol, we test the performance of the algorithm in verifying all the 90 satisfiable instances. Figure S7a shows the detailed results when verifying the satisfiable instances. For all the 90 instances Arthur has high probabilities to accept, which confirm the nearly perfect completeness of the protocol. In addition, we also give the results for the 90 cheating cases where Merlins send the states $|\psi_{\rm ch1}\rangle$ or $|\psi_{\rm ch2}\rangle$ in Fig. S7b. The rejection probabilities for these cheating cases are in the same order of magnitude as the probabilities for verifying satisfiable instances.

Optical swap test. The results of the Hong-Ou-Mandel (HOM) interference shown in the main text are based on the detection events of the 15 two-fold coincidence channels. Here we observe the HOM interference for all the 15 outcomes, as illustrated in Fig. S8. The interferences for the 6 "accept" channels manifest peaks whereas the interferences for the 9 "reject" channels manifest dips. In visualizing the results of the HOM interference in Fig. 4b in the main text, a factor of 3/2 is applied to the probability of the "accept" outcome to compensate the events that two photons trigger the same detector. To demonstrate the detection of events that two photons are in the same path, we also implement the optical swap test with additional detectors, as depicted in Fig. S9. The additional detectors add photon-number resolution to two of the optical paths. Under this detection scheme, we perform the optical swap tests on states that are the same as in the main text. For the case that the two states are the same, higher acceptance probability is observed compared the results with 6 detectors.

III. AMPLIFICATION OF THE SUCCESS PROBABILITY

For practical use of verification algorithms, a problem of particular interest is the ability to amplify the success probability of Arthur's decision. It has been conjectured and proven that any QMA(2) protocol can be amplified

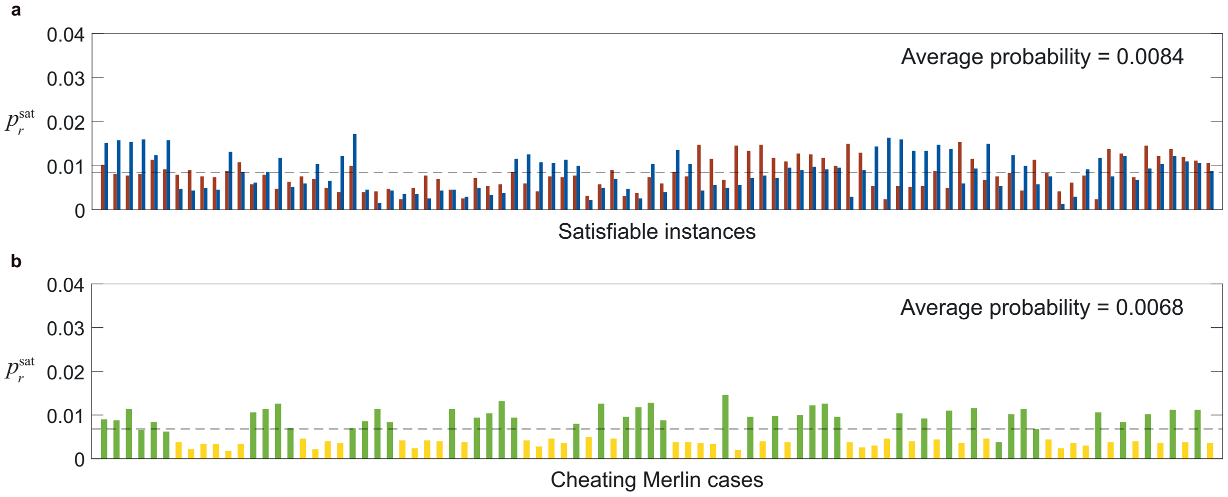

FIG. S7. (a) The rejection probabilities of the satisfiability test for all the 90 satisfiable instances. For each instance, there are two satisfying assignments (proof states), which are denoted as red bars and blue bars respectively. (b) The rejection probabilities of the satisfiability test for the cheating cases. In each case, Merlins send $|\psi_{\rm ch1}\rangle$ (yellow bars) or $|\psi_{\rm ch2}\rangle$ (green bars) as proof states.

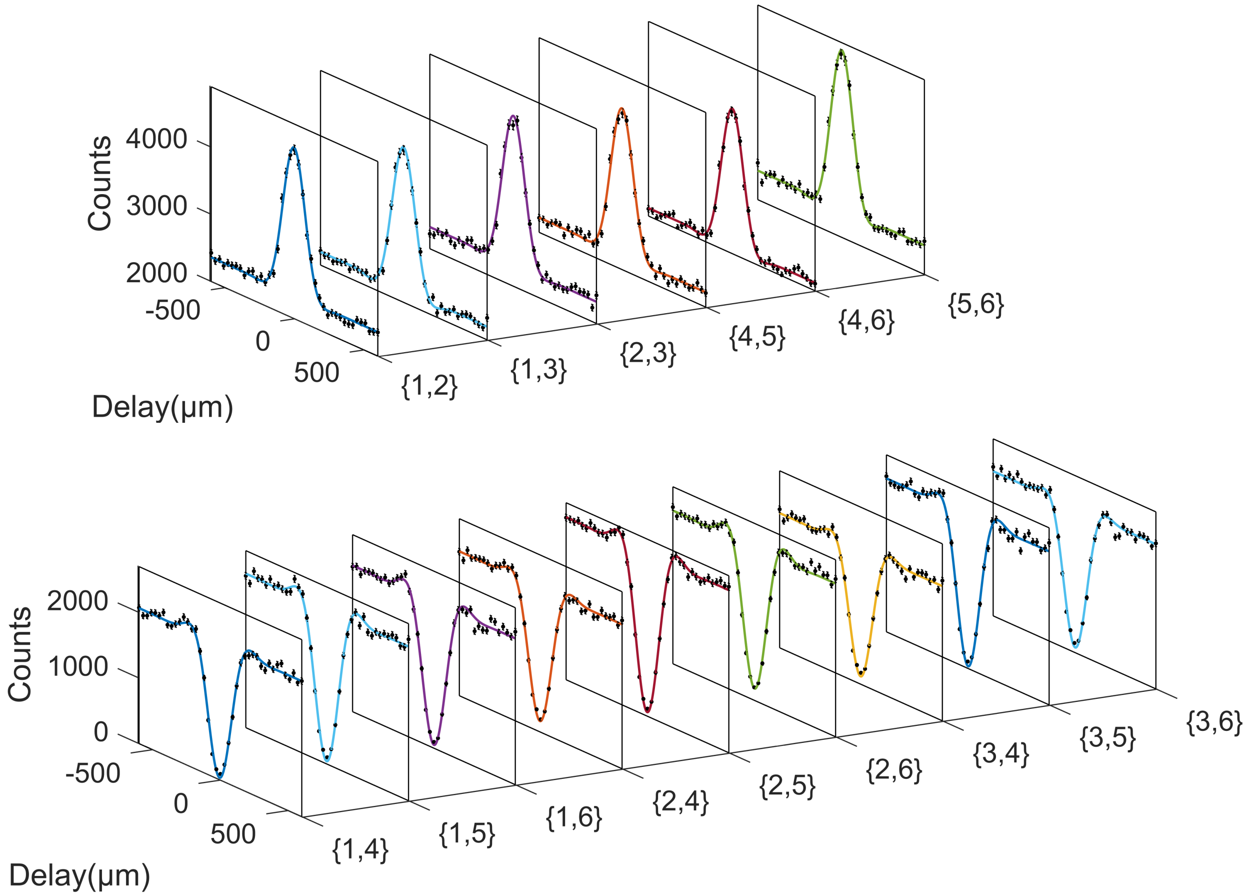

FIG. S8. Observation of Hong-Ou-Mandel (HOM) interference for the 15 coincidence channels. There are 6 one-side channels corresponding to the "accept" output (the upper panel) and 9 two-side channels corresponding to the "reject" output (the lower panel). Solid lines are curve fittings of the data (black dots) to a Gaussian multiplied by sinc function. Error bars are uncertainties assuming Poisson count statistics.

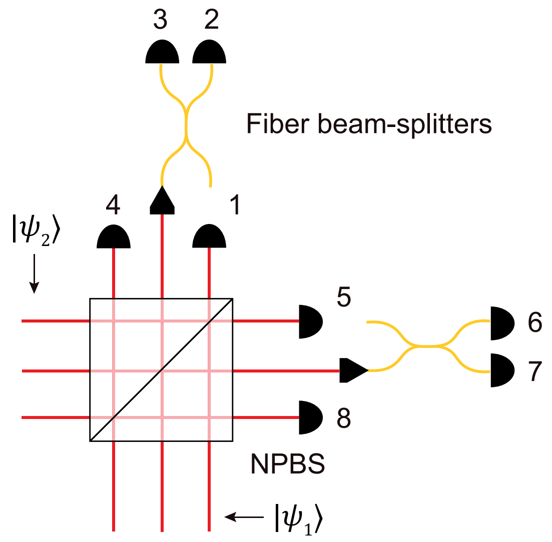

FIG. S9. Optical swap test with additional detectors. The HOM interference scheme is as described in the main text (Fig. 4a). Two 50:50 fiber beam-splitters are attached to the middle optical path in the two output sides respectively. Additional SPADs are also coupled to the outputs of the fiber beam-splitters. The two-fold coincidences for the clicks of the 8 detectors are registered.

to exponentially small error [1, 2]. Here we resort to an amplification protocol proposed in Ref. [1] to demonstrate the amplification of the success probability. The main idea is to repeat the original verification protocol a certain number of times and then output an answer based on specific criteria. For example, Merlins and Arthur firstly run the verification protocol T times, then Arthur accepts if at least $(c+s)T/2$ runs of the verification algorithm output "accept" and rejects otherwise. Here c and s represent completeness and soundness of the original protocol respectively. As an example, we perform the amplification protocol in verifying the satisfiability of instances ϕ_1 and ϕ_2 used in the main text. The success probability of the verification increases with the increase of the number of repetition, as shown in Fig. S11.

[1] Aaronson, S., Beigi, S., Drucker, A., Fefferman, B. & Shor, P. The power of unentanglement. *Theory of Computing* **5**, 1–42 (2009).
[2] Harrow, A. W. & Montanaro, A. Testing product states, quantum Merlin-Arthur games and tensor optimization. *J. ACM* **60**, 3:1–3:43 (2013).

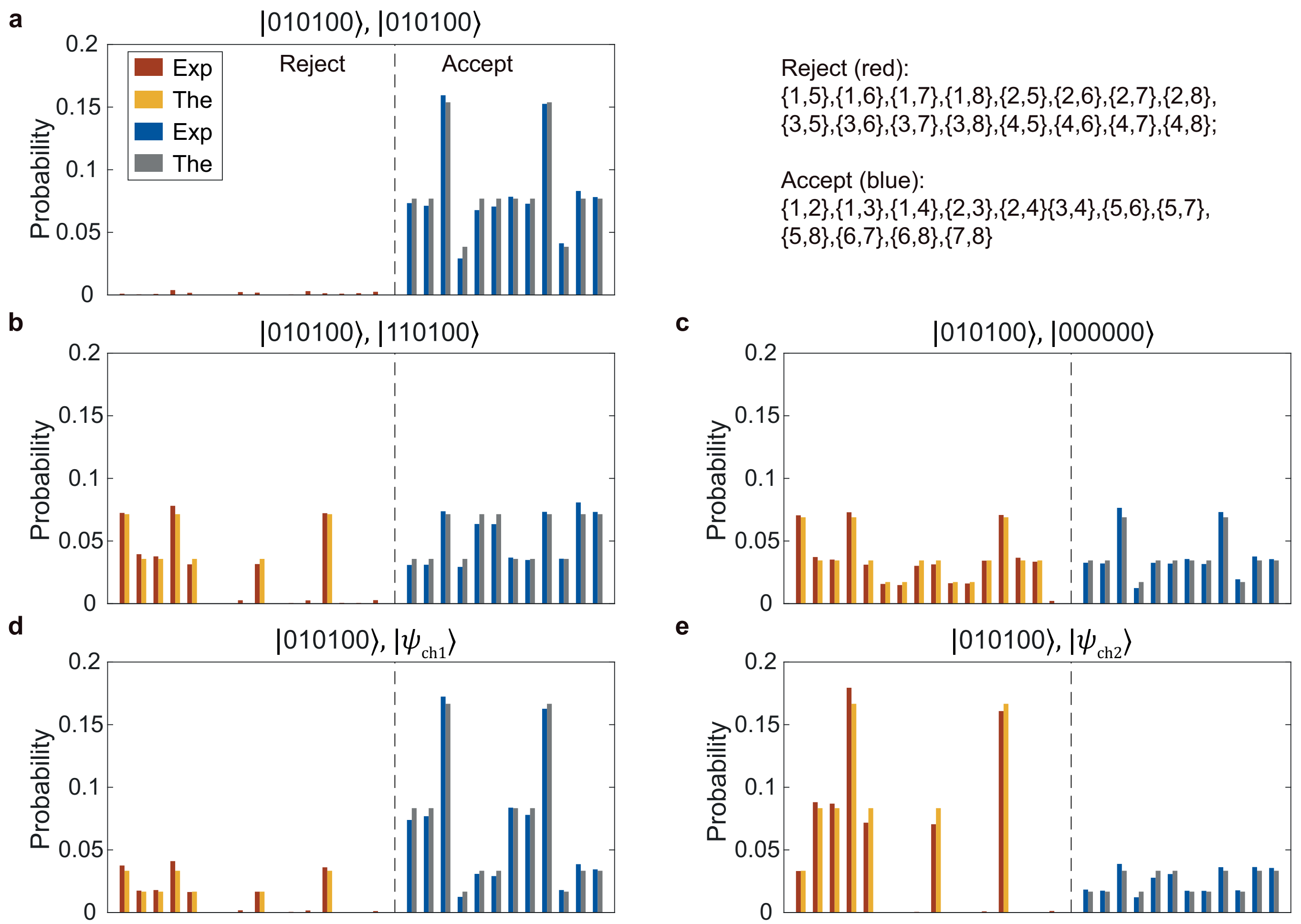

FIG. S10. The results of optical swap test with additional detectors for typical cases: (a) the two states are proper and identical; (b,c) the two states are proper but not identical, (d,e) one of the state is proper and the another is improper. Each panel shows the experimental (red and blue bars) and theoretical (yellow and grey bars) outcome probabilities on the 28 coincidence channels.

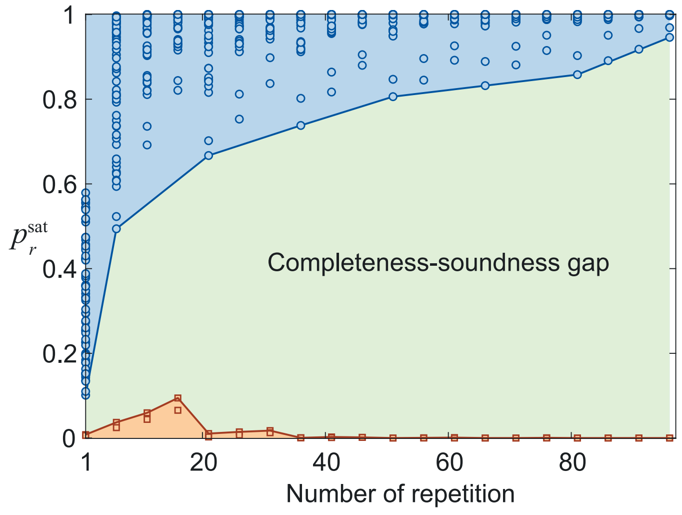

FIG. S11. The amplification of the completeness-soundness gap. Each marker represents the rejection probability of a proof in verifying the example satisfiable instance ϕ_1 (circle, blue) and the example unsatisfiable instance ϕ_2 (square, red) in the main text. The success probability of the verification (green area) increases with the increase of the number of repetition.
